# Comparative Genomics of Rumen *Butyrivibrio* spp. Uncovers a Continuum of Polysaccharide-Degrading Capabilities

**DOI:** 10.1128/AEM.01993-19

**Published:** 2019-12-13

**Authors:** Nikola Palevich, William J. Kelly, Sinead C. Leahy, Stuart Denman, Eric Altermann, Jasna Rakonjac, Graeme T. Attwood

**Affiliations:** a AgResearch Limited, Grasslands Research Centre, Palmerston North, New Zealand; b Donvis Limited, Palmerston North, New Zealand; c Agriculture and Food (CSIRO), St. Lucia, Queensland, Australia; d Institute of Fundamental Sciences, Massey University, Palmerston North, New Zealand; INRS—Institut Armand-Frappier

**Keywords:** rumen, bacteria, polysaccharide, *Butyrivibrio*, *Pseudobutyrivibrio*, genome, CAZy, enolase

## Abstract

Plant polysaccharide breakdown by microbes in the rumen is fundamental to digestion in ruminant livestock. Bacterial species belonging to the rumen genera *Butyrivibrio* and *Pseudobutyrivibrio* are important degraders and utilizers of lignocellulosic plant material. These bacteria degrade polysaccharides and ferment the released monosaccharides to yield short-chain fatty acids that are used by the ruminant for growth and the production of meat, milk, and fiber products. Although rumen *Butyrivibrio* and *Pseudobutyrivibrio* species are regarded as common rumen inhabitants, their polysaccharide-degrading and carbohydrate-utilizing enzymes are not well understood. In this study, we analyzed the genomes of 40 *Butyrivibrio* and 6 *Pseudobutyrivibrio* strains isolated from the plant-adherent fraction of New Zealand dairy cows to explore the polysaccharide-degrading potential of these important rumen bacteria. Comparative genome analyses combined with phylogenetic analysis of their 16S rRNA genes and short-chain fatty acid production patterns provide insight into the genomic diversity and physiology of these bacteria and divide *Butyrivibrio* into 3 species clusters. Rumen *Butyrivibrio* bacteria were found to encode a large and diverse spectrum of degradative carbohydrate-active enzymes (CAZymes) and binding proteins. In total, 4,421 glycoside hydrolases (GHs), 1,283 carbohydrate esterases (CEs), 110 polysaccharide lyases (PLs), 3,605 glycosyltransferases (GTs), and 1,706 carbohydrate-binding protein modules (CBM) with predicted activities involved in the depolymerization and transport of the insoluble plant polysaccharides were identified. *Butyrivibrio* genomes had similar patterns of CAZyme families but varied greatly in the number of genes within each category in the Carbohydrate-Active Enzymes database (CAZy), suggesting some level of functional redundancy. These results suggest that rumen *Butyrivibrio* species occupy similar niches but apply different degradation strategies to be able to coexist in the rumen.

**IMPORTANCE** Feeding a global population of 8 billion people and climate change are the primary challenges facing agriculture today. Ruminant livestock are important food-producing animals, and maximizing their productivity requires an understanding of their digestive systems and the roles played by rumen microbes in plant polysaccharide degradation. Members of the genera *Butyrivibrio* and *Pseudobutyrivibrio* are a phylogenetically diverse group of bacteria and are commonly found in the rumen, where they are a substantial source of polysaccharide-degrading enzymes for the depolymerization of lignocellulosic material. Our findings have highlighted the immense enzymatic machinery of *Butyrivibrio* and *Pseudobutyrivibrio* species for the degradation of plant fiber, suggesting that these bacteria occupy similar niches but apply different degradation strategies in order to coexist in the competitive rumen environment.

## Abstract (precis)

Feeding a global population of 8 billion people and climate change are the primary challenges facing agriculture today. Ruminant livestock are important food-producing animals, and maximizing their productivity requires an understanding of their digestive systems and the roles played by rumen microbes in plant polysaccharide degradation. Members of the genera *Butyrivibrio* and *Pseudobutyrivibrio* are a phylogenetically diverse group of bacteria and are commonly found in the rumen, where they are a substantial source of polysaccharide-degrading enzymes for the depolymerization of lignocellulosic material. Our findings have highlighted the immense enzymatic machinery of *Butyrivibrio* and *Pseudobutyrivibrio* species for the degradation of plant fiber, suggesting that these bacteria occupy similar niches but apply different degradation strategies in order to coexist in the competitive rumen environment.

## INTRODUCTION

The need to feed a growing global population (1) is driving renewed interest in understanding the role of the rumen microbiota in the degradation and conversion of plant polysaccharides into high-value animal products (2). The rumen is one of the most efficient plant polysaccharide depolymerization and utilization systems known, and its microbes are promising sources of fibrolytic enzymes for application in the production of biofuels from lignocellulosic material (3). Rumen bacteria are responsible for most of the breakdown of plant fiber via close interactions among phylogenetically different, but physiologically complementary, bacterial species (4, 5). Species belonging to the genera *Butyrivibrio* and *Pseudobutyrivibrio* form a significant group of rumen bacteria (6, 7) and are among a small number of rumen microbes capable of utilizing xylans and pectins (8–13). *Butyrivibrio* species contribute to fiber digestion in both animals (14–17) and humans (18) due to their ability to degrade hemicelluloses (19–22) and are also involved in protein breakdown (23) and the biohydrogenation of fatty acids (24, 25). At present, the genus *Butyrivibrio* includes the rumen species Butyrivibrio fibrisolvens, B. hungatei, and B. proteoclasticus and the human species B. crossotus (26–30), while the genus *Pseudobutyrivibrio* has two species, Pseudobutyrivibrio xylanivorans and P. ruminis. Due to the substantial morphological (31), metabolic (32–34), and serological (35, 36) differences, it is likely that more distinct species groups of *Butyrivibrio* and *Pseudobutyrivibrio* exist in the rumen.

*Butyrivibrio* and *Pseudobutyrivibrio* strains encode a more impressive repertoire of carbohydrate-active enzymes (CAZymes) than most *Firmicutes* (7), including those involved in the degradation of pectin (glycoside hydrolase 28 [GH28], polysaccharide lyase 1 [PL1], PL9, PL10, PL11, carbohydrate esterase 8 [CE8], CE12) and xylan (GH8, GH10, GH11, GH43, GH51, GH67, GH115, GH120, GH127, CE1, CE2) (7, 37). Here, we provide a multistrain systematic phenotypic and comparative genomic analysis of rumen *Butyrivibrio* and *Pseudobutyrivibrio* species and show that they are capable of growing on a range of carbohydrates, from simple mono- or oligosaccharides to complex plant polysaccharides, such as pectins, mannans, starch, and hemicelluloses.

(This research was conducted by N. Palevich in partial fulfillment of the requirements for a Ph.D. from Massey University, Manawatu, New Zealand, 2016 [37].)

## RESULTS

### Rumen *Butyrivibrio* strains are phylogenetically diverse.

Phenotypic characterizations, including the characterization of cell morphology, motility, carbon source utilization, and fermentation end products, and genotypic characterizations, including characterization by 16S rRNA gene sequencing and pulsed-field gel electrophoresis (PFGE), were carried out on 30 *Butyrivibrio* strains from the rumen environment. Microscopic evaluation of cells from liquid cultures and from colonies on plates confirmed that each of the 30 *Butyrivibrio* strains displayed morphologies consistent with those of *Butyrivibrio* strains (see Data Set S1 in the supplemental material). Based on analysis of full-length 16S rRNA gene sequences (Fig. S1), all *Butyrivibrio* strains clustered separately from *Pseudobutyrivibrio* strains and grouped into three clusters. Cluster 1 contained the sequences of the type strains of B. proteoclasticus (B316^T^) and B. hungatei (JK615^T^) and 10 other *Butyrivibrio* strains. Cluster 2 contained the sequences of 12 *Butyrivibrio* strains, none of which were type strains, and cluster 3 consisted of the sequences of 8 strains containing the B. fibrisolvens type strain (D1^T^) and closely related strains (Fig. S1). Clusters 2 and 3 were well supported by bootstrap analyses, showing 92% and 97% bootstrap support, respectively, while cluster 1 was more diverse, showing only 57% bootstrap support (Data Set S1). These results suggest that the *Butyrivibrio* 16S rRNA gene sequences can be divided into two relatively cohesive clusters, clusters 2 and 3, while the larger cluster, cluster 1, is a continuum of related sequences containing those of at least two species.

PFGE analyses of genomic DNAs digested with the restriction endonucleases (REs) ApaI and I-CeuI produced unique banding patterns for all of the *Butyrivibrio* strains analyzed, providing evidence for differences at the genomic DNA level between these organisms. The genome size estimates from the RE digests ranged from approximately 3.5 Mb to 5.6 Mb (Data Set S1), with the average size being 4.14 Mb. PFGE analyses of undigested genomic DNAs also identified large extrachromosomal elements, with an average size range of from 300 to 500 kb. The largest extrachromosomal DNA was an 869.2-kb element from *Butyrivibrio* sp. strain XPD2002, and the smallest was a 99.1-kb element from *Butyrivibrio* sp. strain AE3006 (Data Set S1). Comparisons of the draft genomes in regard to the sizes of the extrachromosomal elements identified that two of the six *Butyrivibrio* sp. strain AE3004 contigs matched the 433.1- and 350.1-kb bands observed. B. fibrisolvens FE2007 and WTE3004, as well as *Butyrivibrio* sp. strains AE3006, MB2005, AC2005, LC3010, VCD2006, WCD2001, VCB2006, XBB1001, FCS006, and NC2007, also possessed contigs similar to the PFGE bands observed. Overall, the patterns from the PFGE analysis indicate that extrachromosomal elements are a common genomic characteristic of rumen *Butyrivibrio* species.

**FIG 1:**
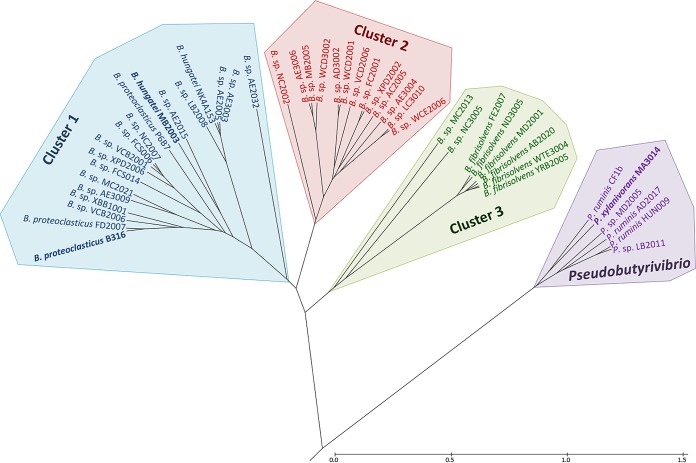
FGD of *Butyrivibrio* (*B*.) and *Pseudobutyrivibrio* (*P*.) genomes. The predicted ORFeomes of all 46 genomes were subjected to an FGD analysis, and the resulting distance matrix was imported into MEGA6 (82). The functional distribution was visualized using the UPGMA method (113, 114). The tree is drawn to scale, with the branch lengths being in the same units as those of the functional distances used to infer the distribution tree. The bar represents the number of nucleotide substitutions per site.

Comparative genome analyses were carried out on the 30 *Butyrivibrio* strains, along with an additional 10 *Butyrivibrio* and 6 *Pseudobutyrivibrio* strains (Data Set S1), using functional genome distribution (FGD), average nucleotide identity (ANI), and alignment fraction (AF) analyses (38, 39). The findings of FGD analysis (Fig. 1) were consistent with the phylogenetic inferences based on the full-length 16S rRNA gene sequence data (Fig. S1). The ANI and AF identities of whole-genome nucleotide sequences between the proposed clusters of rumen *Butyrivibrio* species varied considerably between the genome pairs (Data Set S1). The ANI and AF identities of the 6 *Pseudobutyrivibrio* strains compared with those of the 40 *Butyrivibrio* strains were between 70% and 71% and ∼0.2, respectively. The clustering of the *Butyrivibrio* and *Pseudobutyrivibrio* genomes based on Pfams, COGs, TIGRfams, and KO protein/functional family types (Data Set S1) were also generally consistent with the 16S rRNA gene-based species grouping and genome similarity comparisons.

Although the demarcation between the *Butyrivibrio* and *Pseudobutyrivibrio* genera was distinct, the boundaries between species within each genus were less distinct. In each of the three *Butyrivibrio* clusters, some strains formed clearly separate groupings. For example, *Butyrivibrio* sp. strains MC2013 and NC3005 clustered away from the rest of the cluster 3 B. fibrisolvens strains, and *Butyrivibrio* sp. strains AE3003, AE2005, and LB2008 and B. hungatei NK4A153 were distinct within *Butyrivibrio* cluster 1 (Fig. 1).

### *Butyrivibrio* genomes include a large number of orthologous gene families.

**FIG 2:**
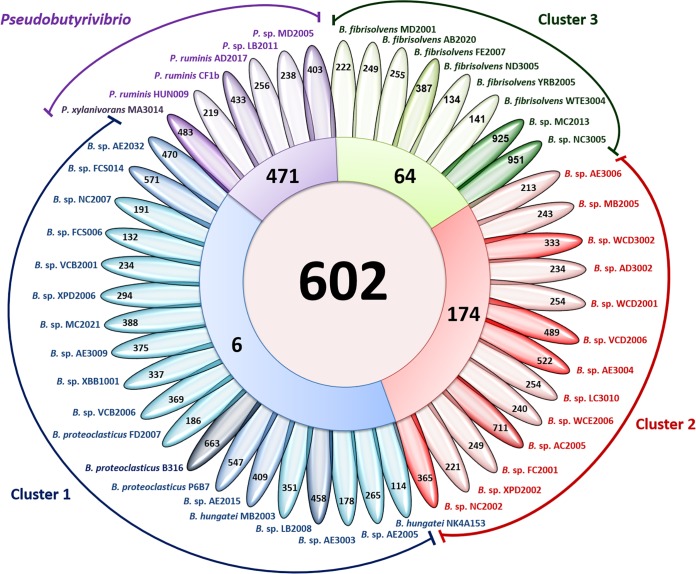
Flower plot diagram of unique, group-specific, and core gene families in the *Butyrivibrio* and *Pseudobutyrivibrio* genomes. The core genome is shown in the center circle. Each colored segment represents the number of gene families shared among the four species groups, and the outer petals represent unique gene families for individual genomes.

The core, variable, and unique gene families present in the *Butyrivibrio* (clusters 1 to 3) and *Pseudobutyrivibrio* genomes were determined using BLAST analyses. A total of 29,105 orthologous gene families were found (Fig. 2 and S2), of which 602 represented the gene families shared among all genomes, or the core genome set. The core genome set consisted mainly of genes encoding housekeeping, carbohydrate metabolism, and transport functions. The *Pseudobutyrivibrio* genomes had the highest number of unique genes (*n* = 471), with predicted functions including flagellum biosynthesis, signal transduction, and the production of acetolactate synthase (ALS), accessory Sec system proteins, diguanylate cyclase (GGDEF), phosphoesterase, cell division protein (FtsA), helicase, β-glucosidase, and GH3 proteins.

Genome alignments using the B. proteoclasticus B316^T^ genome (chromosome, chromid, and plasmids) as the reference (Fig. S3) revealed that only *Butyrivibrio* sp. strain XBB1001, *Butyrivibrio* sp. strain VCB2006, and B. proteoclasticus FD2007 from cluster 1 had significant synteny to B316^T^. FD2007 was the only genome which has a chromid similar to that of the B316^T^ genome. The high similarity of XBB1001, VCB2006, and FD2007 to B316^T^ in the FGD, ANI, and protein family analyses supports their classification as strains of B. proteoclasticus.

The amino acid usgae and codon usage within the predicted proteomes of *Butyrivibrio* and *Pseudobutyrivibrio* were compared (Fig. S4). Isoleucine, leucine, serine, aspartate, glutamate, alanine, lysine, valine, and glycine were the most frequently used amino acids. Codon usage was similar among the *Pseudobutyrivibrio* proteomes, while codon usage varied across the *Butyrivibrio* strains. These findings on codon usage are consistent with those seen among the *Firmicutes*.

### *Butyrivibrio* species are capable of using a wide range of polysaccharides.

Substrate utilization tests and analyses of fermentation end products were carried out to determine the metabolic capacities of the *Butyrivibrio* strains evaluated in this study. There were clear patterns of substrate use by the species groups (Data Set S1). Most strains were able to use glucose, galactose, melezitose, trehalose, glycerol, myoinositol, mannitol, and sorbitol. Cluster 2 and 3 strains were more versatile than cluster 1 strains in terms of soluble carbohydrate utilization. Most *Butyrivibrio* strains were able to use all the substrates tested, apart from arabinose, mannose, rhamnose, maltose, melibiose, xylitol, amygdalin, esculin, rutin, and salicin. Many of the cluster 3 strains and some of the cluster 1 strains were also able to grow on the semisoluble or insoluble substrates pectin and xylan, while none of the strains could use ball-milled cellulose. The B. fibrisolvens MD2001, AB2020, FE2007, ND3005, YRB2005, and WTE3004 strains displayed similar growth patterns, in particular, the ability to utilize pectin, xylan, and xylose, whereas *Butyrivibrio* sp. strains MC2013 and NC3005 expressed different growth patterns that coincided with their phylogenetic and genomic divergence within cluster 3 (Fig. 1). All cluster 2 strains could utilize xylose, and strains AE3004, XPD2002, and VCD2006 displayed similar growth patterns on dextrin, inulin, starch, and xylan. Interestingly, cluster 2 strains were not able to utilize pectin for growth. The cluster 1 strains B316^T^, VCB2006, XBB1001, and AE3009, which grouped closely at the genome and phylogenetic levels, displayed similar growth patterns across all insoluble substrates analyzed, and only AE2015 and B316^T^ could utilize xylose (Data Set S1).

**FIG 3:**
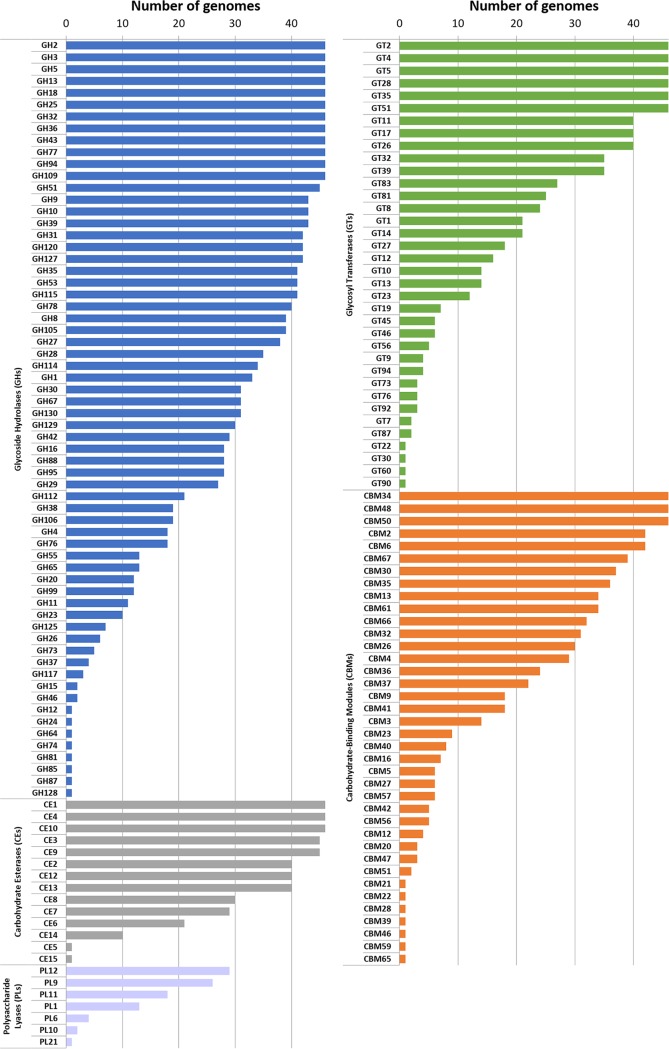
Distribution of each CAZyme class and family in *Butyrivibrio* and *Pseudobutyrivibrio* genomes. Colored bars represent the total numbers of genomes that contain members of the specific CAZyme family present in their genomes.

**FIG 4:**
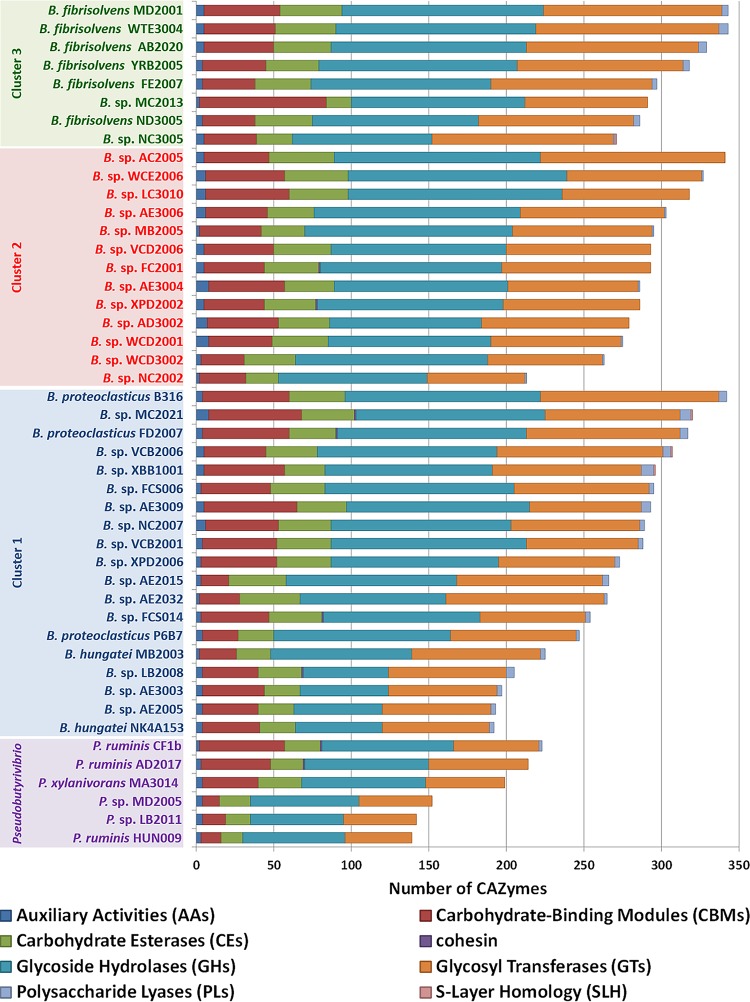
Comparative analysis of annotated *Butyrivibrio* and *Pseudobutyrivibrio* CAZymes. The numbers and types of CAZyme modules or domains are represented as colored horizontal bars.

**FIG 5:**
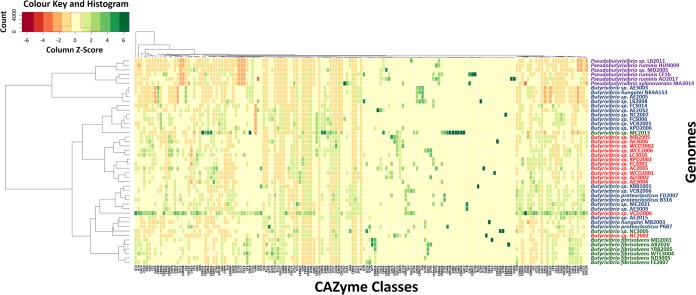
Heat map of normalized relative abundances for CAZyme families determined for the *Butyrivibrio* and *Pseudobutyrivibrio* genomes. The relative normalized inferred CAZy gene family abundances per genome (Z-score) are shown using a heat color scheme (red to green), indicating low to high relative abundance. The Bray-Curtis distances (119) of compositional dissimilarity and hierarchical cluster analysis using Ward’s method (120) were used to calculate normalized abundance. Genome names are colored to represent *Butyrivibrio* cluster 3 in green, *Butyrivibrio* cluster 2 in red, *Butyrivibrio* cluster 1 in blue, and *Pseudobutyrivibrio* in purple.

The polysaccharide-degrading capabilities encoded by the *Butyrivibrio* genomes were further defined using CAZyme analysis. A total of 159 CAZyme families were identified (Fig. 3), consisting of 64 glycoside hydrolases (GHs), 14 carbohydrate esterases (CEs), 7 polysaccharide lyases (PLs), 38 carbohydrate-binding protein modules (CBM), and 36 glycosyltransferases (GTs) (Data Set S2). Within the *Butyrivibrio* species, the strains generally had similar types of CAZymes, but the absolute number of genes within each of their categories in the Carbohydrate-Active Enzymes database (CAZy) varied considerably (Fig. 4; Data Set S2). In particular, B. fibrisolvens strains MD2001, AB2020, YRB2005, and WTE3004 from cluster 3 and cluster 1 strains MC2021, FD2007, VCB2006, XBB1001, FCS006, and AE3009 possessed the largest number of CAZymes within their respective groups (Fig. 4) and grouped together, based on the relative abundance of CAZymes (Fig. 5). Interestingly, the CAZyme cluster analysis indicated that *Butyrivibrio* strain AE3003 and B. hungatei NK4A153, AE2005, and LB2008 clustered most closely with *Pseudobutyrivibrio* strains and well away from their nearest phylogenetic relatives, B. hungatei MB2003 and B. proteoclasticus. Strains MC2013 and VCD2006 and, to a lesser extent, strain NC2002, also had CAZyme profiles atypical of those of their closest relatives and were separated by CAZyme analysis.

Pfam domain analysis of the most abundant GH families (GH2, GH31, GH3, GH13, and GH43) showed that most did not contain signal sequences and, hence, were predicted to be located intracellularly. Similarly, CAZymes with predicted roles in xylan and pectin degradation (the GH8, GH28, GH39, GH51, GH67, GH88, GH105, GH115, CE2, and CE10 families) were also predicted to be intracellular (Data Set S2), suggesting that a variety of complex oligosaccharides resulting from extracellular hydrolysis are transported and metabolized within the cell.

### Fermentation pathways and enolase gene loss.

**FIG 6:**
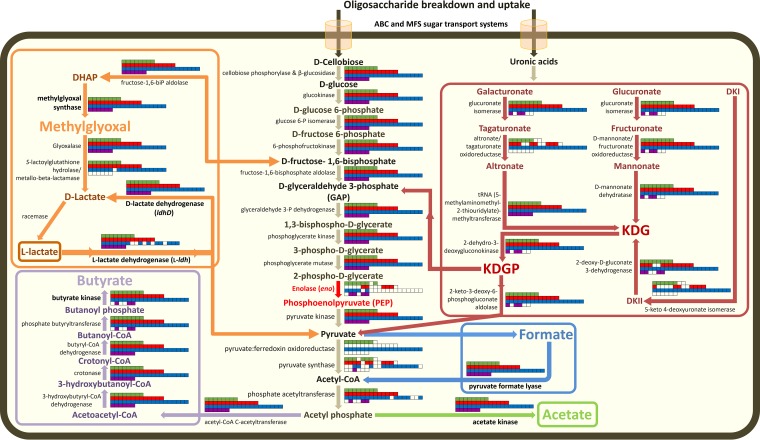
Comparisons of gene presence/absence for enzymes involved in the carbohydrate metabolic pathways in *Butyrivibrio* leading to the formation of butyrate, formate, acetate, and lactate. All metabolic pathways were compiled using information from the MetaCyc (121) and KEGG (122) databases. The presence or absence of genes encoding particular enzymes within genomes is indicated by full or empty cells, respectively in the panels. The order of genomes in the panels, from left to right, are as follows: row 1, *Butyrivibrio* cluster 3 (green) strains AB2020, FE2007, MD2001, ND3005, WTE3004, YRB2005, MC2013, and NC3005; row 2, *Butyrivibrio* cluster 2 (red) strains AE3006, MB2005, WCD3002, AD3002, WCD2001, VCD2006, AE3004, LC3010, WCE2006, AC2005, FC2001, XPD2002, and NC2002; row 3, *Butyrivibrio* cluster 1 (blue) strains NK4A153, AE2005, AE3003, LB2008, MB2003, AE2015, P6B7, B316^T^, FD2007, VCB2006, XBB1001, AE3009, MC2021, XPD2006, VCB2001, FCS006, NC2007, FCS014, and AE2032; and row 4, *Pseudobutyrivibrio* (purple) strains MA3014, HUN009, CF1b, AD2017, LB2011, and MD2005. The enolase-catalyzed reaction is shown in red, as the gene was absent from a number of *Butyrivibrio* strains. Color schemes for the metabolism pathways are as follows: the formation of formate in blue, acetate in green, butyrate in purple, l-lactate in red, and d-lactate by the proposed methylglyoxal shunt in orange (69). Abbreviations: DHAP, dihydroxyacetone phosphate; DKI, 5-keto-4-deoxyuronate; DKII, 2,5-diketo3-deoxygluconate; KDG, 2-keto-3-deoxygluconate; KDGP, 2-keto-3-deoxy-gluconate phosphate. Abbreviations for sugar transport systems are as follows: ABC, ATP binding cassette; MFS, major facilitator superfamily.

The fermentation pathways in *Butyrivibrio* predicted from gene content and metabolic pathway reconstruction are shown in Fig. 6. Genome analysis and metabolic pathway reconstruction of *Butyrivibrio* strains revealed that 7 of the 8 cluster 3 strains (all except MC2013), cluster 2 strains WCD2001, VCD2006, AC2005, FC2001, and XPD2002, and cluster 1 strain AE2015 had all of the genes encoding the enzymes required for fermenting hexoses through to pyruvate via an intact Embden-Meyerhof-Parnas (EMP) pathway. However, 18 *Butyrivibrio* genomes lack an identifiable enolase gene (*eno*), which encodes the enzymatic conversion of 2-phospho-d-glycerate to phosphoenol pyruvate (EC 4.2.1.11) in the second-to-last step of the EMP pathway. Because these genomes are not fully closed, it is possible that the unsequenced regions contain the missing *eno* genes; therefore, genomic DNA from each strain was screened in PCRs using primers specific for *eno* genes. The PCR screens produced an *eno* amplicon in 8 *Butyrivibrio* strains but failed to produce an amplicon in 24 strains (19 putatively *eno*-negative strains and 5 predicted *eno*-positive strains [strains NC3005, WCD2001, VCD2006, AC2005, and AE2015]) (Data Set S2). All *eno*-positive, PCR-positive strains had strong alignments of both the forward and reverse primer sequences with the sequences of their encoded *eno* genes (Fig. S5); however, the 5 *eno*-positive, PCR-negative strains did not. Analysis of the Eno_N (PF03952) and Eno_C (PF00113) Pfam domains of the predicted enolase proteins showed that all cluster 3 strains (except for strain MC2013, which does not have an *eno* gene) and cluster 2 strains FC2001 and WCD2001 contain both the N- and C-terminal domains and are indicated to be full length (432 amino acids [aa]). However, cluster 2 strains AC2005 and VCD2006 and cluster 1 strain AE2015 showed truncated N-terminal domains, while cluster 2 strain XPD2002 had two predicted enolases. The first of these possessed truncated N- and C-terminal domains, and the second contained only the C-terminal Pfam domain with a predicted protein size of 129 aa. The truncated *eno* genes in cluster 2 strains AC2005 and VCD2006 and cluster 1 strain AE2015 explain the lack of *eno* PCR products from these strains. The absence of an *eno* PCR product for B. fibrisolvens NC3005 and cluster 2 strain WCD2001 is explained by an altered *eno* PCR primer site at the 5′ and 3′ ends of these genes (Fig. S5B). The positive *eno* PCR result for XPD2002 is due to the *eno* primer site remaining intact in the shortened gene (129 aa).

The *Butyrivibrio eno*-negative strains and, possibly, the strains containing truncated *eno* genes must use an alternative pathway for hexose metabolism. The methylglyoxal shunt is the most likely alternative pathway, which is mediated by the enzymes fructose-1,6-bisphophate aldolase (Fbp), methylglyoxal synthase (MgsA), glyoxylase (GloA/B), *S*-lactoylglutathione hydrolase, and d-2-hydroxyacid dehydrogenase (LdhD), converting d-fructose-1,6-bisphophate to pyruvate (Fig. 6). The genes encoding these key methylglyoxal shunt enzymes were compared between the genomes of the *Butyrivibrio eno*-positive and *eno*-negative strains (Fig. 6; Data Set S2). Analysis of the fermentation end products identified the production of lactate in *Butyrivibrio* strains (Data Set S1), which was exclusively l-lactate. Of particular interest was *eno*-negative *Butyrivibrio* sp. LC3010, which produced substantial amounts of l-lactate as a fermentation product but which also contained an incomplete set of methylglyoxal shunt pathway genes, in particular, glyoxalase I or lactoglutathione lyase (*gloA*) (Fig. 6; Data Set S2). *Butyrivibrio* sp. LC3010 and other such *Butyrivibrio* strains may thus use alternative enzymes to the methylglyoxal shunt that are yet to be characterized. The genes encoding lactate dehydrogenase (*ldh*) have been identified and compared in the *Butyrivibrio* draft genome sequences (Fig. 6), in which the *ldh* gene encoding l-lactate dehydrogenase plays a key role in the production of l-lactate from pyruvate.

An alternative explanation for the lack of *eno* genes is that *Butyrivibrio* species may be specialized pectin fermenters in the rumen. Pectin breakdown releases galacturonates and glucuronates, which are metabolized via 2-keto-3-deoxygluconate (KDG) rather than via the EMP pathway (Fig. 6). KDG is then converted to 2-keto-3-deoxygluconate phosphate (KDGP) by 2-dehydro-3-deoxygluconokinase and is then converted to pyruvate and glyceraldehyde-3-phosphate (GAP) by 2-keto-3-deoxygluconate 6-phosphate aldolase (40–42). All *Butyrivibrio* genomes encode the enzymes required to convert glucuronate through to pyruvate (Fig. 6). Most strains also encode all the enzymes for galacturonate fermentation through to pyruvate. The only step that is missing in cluster 3 *Butyrivibrio* sp. strains MC2013 and NC3005 and cluster 2 strains AD3002, WCD2001, LC3010, WCE2006, and NC2002 is the tagaturonate conversion to altronate via the altronate/tagaturonate oxidoreductase enzyme. This pathway is not known to generate ATP via electron transport phosphorylation (ETP), but it generates pyruvate, which potentially can lead to ATP production via the pathways described in Fig. 6.

## DISCUSSION

Members of the genus *Butyrivibrio* are a major component of the ruminal microflora and have been isolated from the gastrointestinal tracts and feces of various ruminants, monogastric animals, and humans (13–16, 21–25, 43, 44). Until 1996, all *Butyrivibrio* and *Pseudobutyrivibrio* strains were assigned to a single species, Butyrivibrio fibrisolvens, due to their phenotypic and metabolic similarities (8). Rumen *Butyrivibrio* and *Pseudobutyrivibrio* strains are currently divided into six species, represented by B. fibrisolvens, B. hungatei, B. proteoclasticus and B. crossotus and the *Pseudobutyrivibrio* species P. xylanivorans and P. ruminis. All of these species belong to the genetically diverse *Lachnospiraceae* family, within the order *Clostridiales* (26). Based on 16S rRNA gene sequences, the *Butyrivibrio* strains grouped into three clusters, designated *Butyrivibrio* cluster 1, containing B. proteoclasticus (B316^T^), B. hungatei (JK615^T^), and 10 other *Butyrivibrio* strains; cluster 2, containing 12 *Butyrivibrio* strains; and cluster 3, containing 8 strains, including the B. fibrisolvens type strain (D1^T^). Clusters 2 and 3 are each phylogenetically cohesive, while the larger cluster, cluster 1, appears to contain a continuum of related organisms containing at least two species. The FDG analyses gave phylogenetic associations consistent with the 16S rRNA gene sequence data, while the ANI and AF identities of whole-genome nucleotide sequences between the proposed clusters of rumen *Butyrivibrio* strains varied considerably.

It is apparent that while the *Butyrivibrio* and *Pseudobutyrivibrio* genomes share about 600 core genes, they also carry unique selections of genes drawn from the species’ accessory genomes. Compared to *Prevotella* species from different sites in humans, *Butyrivibrio* species have a large number of orthologous gene families (45). Recent work has shown that genes are gained and lost through the combined actions of gene loss, gene gain via lateral transfer, and gene duplication at higher rates in organisms on the tips of the phylogenetic tree (46, 47). Examples of *Butyrivibrio* species that may exhibit such plasticity at the genome level include *Butyrivibrio* cluster 1 strains B. proteoclasticus P6B7 and *Butyrivibrio* sp. strains LB2008 and AE2032, cluster 2 strain *Butyrivibrio* sp. strain NC2002, and cluster 3 strains *Butyrivibrio* sp. MC2013 and NC3005. This pattern could fit the scenario that among *Butyrivibrio* species accessory genes are transiently advantageous in only a small subset of strains.

The collective genome complement (29,105 genes) and the core genome (602 genes) of all rumen *Butyrivibrio* and *Pseudobutyrivibrio* strains reflect a large reservoir of genetic diversity within this group (Fig. 2). The strict core genome represents 2% of the collective genome and represents the proposed minimum set of genes that allow the survival of *Butyrivibrio* species in the rumen, including genes encoding protein processing, folding and secretion (predominantly translation, including ribosome function, maturation, modification, protein turnover, and RNA degradation), cellular processes (cell division and transport), and energy and metabolism (lipid metabolism and biosynthesis of nucleotides and cofactors) and numerous poorly characterized genes (conserved hypothetical proteins, etc.). The size of the collective *Butyrivibrio* genome increased with the number of genomes analyzed due to unique strain-specific genes and reflects the ability of this group of organisms to occupy different niches within the rumen environment.

Early studies reported the presence of large extrachromosomal elements in a number of *Butyrivibrio* strains (48), and recently, megaplasmids and chromids were described for B. proteoclasticus B316^T^ and B. hungatei MB2003 (49–53). The present study has confirmed that extrachromosomal chromids and megaplasmids are common in *Butyrivibrio* species and possibly improve their competitiveness by increasing the number of genes carried in the bacterial genome through gene dosing effects and allow for faster genome replication and a higher growth rate of the bacterial cell (54). A rumen plasmidome study identified phylogenetic associations of various plasmid genes across taxonomic levels up to the phylum level, emphasizing the essential evolutionary and cooperative roles between plasmids and their host bacteria (55). Due to the incidence of related plasmids in phylogenetically distant bacteria, coupled with the ability to be horizontally transferred by conjugation, plasmids likely play a role as a channel for the horizontal exchange of genomic material, conveying advantageous functions between the rumen microbes. The types of traits that are transferred by plasmids include those implicated in amino acid, protein, and carbohydrate metabolism, which are essential in the rumen ecosystem, and make the metabolic burden of sustaining the plasmid worthwhile for the host (56). The transfer of genes encoding degradative systems (57), exopolysaccharide production (58), bacteriocin production (59, 60), and resistance to antibiotics (61, 62) may also provide a competitive advantage within the rumen microbial ecosystem. In *Butyrivibrio*, it is possible that extrachromosomal elements serve as vehicles for the exchange of genomic information between different strains and species and potentially to other genera, such as *Pseudobutyrivibrio*.

Early characterizations of *Butyrivibrio* strains indicated that some had the ability to degrade cellulose and that most strains were able to digest xylan and pectin substrates (8, 10). Later work concluded that glucose, cellobiose, maltose, and esculin were universally used substrates of *Butyrivibrio* strains (30). Rumen *Butyrivibrio* and *Pseudobutyrivibrio* strains are reported to use a wide range of soluble and some insoluble substrates and characteristically ferment carbohydrates to butyrate, formate, lactate, and acetate (26, 30). The findings from the present study show the absence of growth on cellulose by all 30 *Butyrivibrio* strains as well as P. xylanivorans MA3014. The volatile fatty acid (VFA) production data support the notion that rumen *Butyrivibrio* strains are metabolically versatile and can utilize a wide range of insoluble substrates but are not cellulose-degrading bacteria. The substrate utilization patterns of the *Butyrivibrio* species generally followed the groupings defined by the phylogenetic and genomic analyses. For example, the B. fibrisolvens MD2001, AB2020, FE2007, ND3005, YRB2005, and WTE3004 strains displayed similar growth patterns, in particular, their ability to utilize pectin, xylan, and xylose, whereas cluster 3 strains MC2013 and NC3005 expressed different growth patterns that coincided with their phylogenetic and genomic divergence within this cluster (Fig. 1). All cluster 2 strains could utilize xylose, and strains AE3004, XPD2002, and VCD2006 displayed similar growth patterns on all insoluble substrates analyzed. Cluster 1 strains B316^T^, VCB2006, XBB1001, and AE3009, which grouped closely at the genome and phylogenetic levels, also displayed similar growth patterns across all insoluble substrates (except cellulose) and monosaccharides analyzed.

The comparative genome analyses identified considerable variation in the conservation of orthologous gene families both between and within the rumen *Butyrivibrio* strains. This suggests a degree of specialization within these bacteria, in addition to the presence of a set of genes required for polysaccharide degradation in the rumen. The analyses of the *Butyrivibrio* and *Pseudobutyrivibrio* CAZymes involved in the breakdown of complex carbohydrates showed variation in their distribution and abundance. The abundance of GH, PL, and CE domain-containing CAZymes encoded within the genomes of the cluster 1 and 2 strains suggests that they are specialist degraders of xylan and pectin (see Data Set S2 in the supplemental material). This suggests that the members of cluster 1 play an important role in polysaccharide degradation in the rumen and are significant energy suppliers to ruminants through the production of VFAs. The growth experiments and comparative glycobiome analyses have shown that strains belonging to this species group are diverse in both the substrates that they can utilize and the set of CAZymes that they encode and may occupy similar niches in the rumen.

A previous analysis of the B. proteoclasticus B316^T^ genome described that two-thirds of its CAZymes involved in polysaccharide breakdown were predicted to be intracellular and that only a few CBMs were present (49), and this was also true for the strains in this study. These observations indicate that the ability to degrade plant fiber and utilize the released carbohydrates for growth are important features defining the differences between *Butyrivibrio* strains and may reflect how these strains occupy different niches within the rumen in order to coexist. Investigations of the xylan and pectin utilization abilities of B. hungatei MB2003 and B. proteoclasticus B316^T^ in coculture support this view (63). In monocultures, B316^T^ was able to grow well on xylan and pectin, while MB2003 was unable to utilize either of these insoluble substrates to support significant growth. Cocultures of B316^T^ grown with MB2003 revealed that MB2003 showed growth almost equivalent to that of B316^T^ when either xylan or pectin was supplied as the substrate. The effect of coculture on the transcriptomes of B316^T^ and MB2003 was assessed, where B316^T^ transcription was largely unaffected by the presence of MB2003. However, MB2003 expressed a wide range of genes encoding proteins for carbohydrate degradation, central metabolism, oligosaccharide transport, and substrate assimilation, in order to compete with B316^T^ for the released sugars. These results suggest that B316^T^ has a role as an initiator of the primary solubilization of xylan and pectin, while MB2003 competes effectively for the released soluble sugars to enable its growth and maintenance in the rumen.

*Butyrivibrio* and *Pseudobutyrivibrio* have previously been grouped into lactate-producing and lactate-nonproducing strains (30). Also, the diversity in fermentation products observed within each *Butyrivibrio* cluster suggests differences in the metabolic pathways of each strain and further highlights the importance and adaptive role of *Butyrivibrio* in the digestion of fibrous constituents of animal feed. The present study has shown an ability of *Butyrivibrio* as a genus to utilize a wide variety of substrates, especially xylan and pectin, suggesting that these microorganisms play an important role in hemicellulose and pectin degradation in the rumen. In particular, the production of large amounts of VFAs by B. proteoclasticus B316^T^ and *Butyrivibrio* sp. strain AE3009 on a range of insoluble substrates indicates the ability of certain *Butyrivibrio* to switch substrate utilization from the simple cellobiose substrate to insoluble polysaccharides in monoculture. Therefore, it is hypothesized that certain *Butyrivibrio* strains are unable to initiate significant degradation of the insoluble polysaccharides alone and rely on more specialized *Butyrivibrio* species to initiate the degradation process, resulting in the release of soluble sugars, for which they compete.

Given the important role of rumen *Butyrivibrio* strains in plant fiber degradation (6, 7, 63), genome sequence information was used to analyze their collective and individual polysaccharide-degrading potential. There are examples of contrasting differences in the abundance of some GH families in *Butyrivibrio* versus *Pseudobutyrivibrio*. For instance, *Butyrivibrio* strains have many members of GHs 28, 30, 38, 65, 67, 88, 105, 112, and 129 (Data Set S2), with predicted activities such as polygalacturonases, β-xylosidases, α-mannosidases, α-trehalases, α-glucuronidases, β-glucuronyl hydrolases, rhamnogalacturonyl hydrolases, lacto-*N*-biose phosphorylases, and α-*N*-acetylgalactosaminidases, respectively (Data Set S2). In contrast, these were completely absent from the six *Pseudobutyrivibrio* strains. Analysis of the CAZy profiles of the rumen *Butyrivibrio* species presented here suggests that, due to their extensive repertoire of GH domain-containing CAZymes, *Butyrivibrio* cluster 1 and 3 strains rather than *Pseudobutyrivibrio* strains are the predominant degraders of xylan and pectin.

The carbon source utilization and VFA data combined with genome similarity and CAZyme analyses showed that *Butyrivibrio* utilization of polysaccharides and the ability of the *Butyrivibrio* strains to assimilate the degradation products of these polysaccharides were variable, with clear differences being identified between species groups based on their initial phylogenetic placements. For the production of butyrate and H_2_ from glucose, rumen *Butyrivibrio* genomes possess a pyruvate:ferredoxin oxidoreductase gene (*nifJ*) required for pyruvate conversion to acetyl coenzyme A (CoA), as well as a butyryl-CoA dehydrogenase/electron-transferring flavoprotein (encoded by *bcd-etfAB*) to generate ATP by classic substrate-level phosphorylation (SLP). In addition, an alternative pathway exists where formate is predicted to be the end product and involves the decarboxylation of acetyl-CoA by a pyruvate formate lyase (encoded by *pflB*) instead of NifJ. It has been proposed that Ech and Rnf work in concert with NifJ and the Bcd-Etf complex to drive ATP synthesis by ETP during glucose fermentation to butyrate (64–66). Interestingly, the vast majority of anaerobic prokaryotes appear to possess either an Ech or an Rnf protein but not both (67, 68). However, a recent analysis of rumen prokaryotic genomes identified rumen *Butyrivibrio* species to be a rare group of bacteria that possess genes for both Ech and Rnf. These findings warrant further biochemical investigation to determine the activity of Ech and Rnf in *Butyrivibrio*.

In ruminal anaerobes, hexoses are usually fermented via the Embden-Meyerhof-Parnas (EMP) glycolytic pathway to pyruvate and from pyruvate to a variety of end products, depending on the organism. In *Butyrivibrio* species, these end products are principally formate and butyrate, with a small amount of acetate, while some strains are also capable of producing lactate. The rumen *Butyrivibrio* pathways for butyrate production presume the possession of a complete EMP glycolytic pathway (Fig. 6). Enolase (encoded by *eno*; EC 4.2.1.11) converts 2-phospho-d-glycerate to phosphoenolpyruvate in the second-to-last step of the EMP pathway. In the proposed alternative methylglyoxal shunt pathway (49), the dihydroxyacetone phosphate (DHAP) is transformed to pyruvate via methylglyoxal and d-lactate dehydrogenase, encoded by *ldhD* (69). The rumen *Butyrivibrio* genomes presented here have the same set of genes previously reported for MB2003 (52, 63) and B316^T^ (49) for the production of butyrate, formate, acetate, and lactate, and these genes appear to be common features among these rumen organisms.

*Butyrivibrio* and *Pseudobutyrivibrio* species form a significant group of rumen bacteria that play an important role in the carbon flow within the rumen by initiating the breakdown of lignocellulose and metabolizing the by-products to short-chain fatty acids and fermentation end products. *Butyrivibrio* and *Pseudobutyrivibrio* strains encode a large and diverse spectrum of degradative CAZymes and binding proteins. In total, 4,421 GHs, 1,283 CEs, 110 PLs, 3,605 GTs, and 1,706 CBMs with predicted activities involved in the depolymerization and utilization of the insoluble plant polysaccharides, such as xylan and pectin, were identified. The different *Butyrivibrio* species were found to possess similar CAZyme repertoires, but with variations in the absolute number of genes within each CAZy category. This apparent functional redundancy encoded by closely related strains was also observed with examination of both 16S rRNA marker gene and genome sequence-based species group demarcations. Herein, a significant example of gene loss was highlighted by the absence of an identifiable enolase, a key enzyme that drives the penultimate step of glycolysis, in the majority of *Butyrivibrio* strains. The comparative genome analyses provide further evidence for the need to include genome sequencing as a prerequisite for the description of new species of bacterial isolates. Together with previous gene expression data, our findings suggest that members of the genera *Butyrivibrio* and *Pseudobutyrivibrio* occupy similar niches but apply different degradation strategies within the rumen.

## MATERIALS AND METHODS

### Cultures used in this study and growth conditions.

The full list of cultures, their provenance, and the phenotypic characteristics for a selection of the *Butyrivibrio* isolates used in the project are shown in Data Set S1 in the supplemental material. The bacterial cultures used in this study were grown as previously described (37, 70). New Zealand *Butyrivibrio* and *Pseudobutyrivibrio* cultures from the Hungate Collection are available from the AgResearch culture collection (7).

### Bacterial genomic DNA isolation and identification.

Genomic DNA was extracted using a Qiagen Genomic-tip kit following the manufacturer’s instructions for the 500/G size extraction. Extracted DNA was stored at −80°C until required. Purified DNA was subject to partial 16S rRNA gene sequencing to confirm strain identity, before being shipped to the U.S. Department of Energy (DOE) Joint Genome Institute (JGI), USA, for sequencing.

### Phylogenetic analysis of full-length 16S rRNA gene sequences.

To determine the phylogenetic relationships of the *Butyrivibrio* isolates shown in Fig. S1, the extracted DNA was PCR amplified using the primer pair fD1 (5′-GAGTTTGATCMTGGCTCAG-3′) and rD1 (5′-AAGGAGGTGATCCARCCG-3′) to amplify the V1-V3 region of the 16S rRNA gene. The PCR cycling conditions used were 94°C for 2 min, followed by 35 cycles of 94°C for 30 s, 56°C for 30 s, and 72°C for 2 min and a final extension time of 10 min at 72°C. The full-length 16S rRNA marker gene sequences (>1,400 bp) obtained by PCR amplification were Sanger sequenced using eight primers: fD1, rD1, 1492r, 1382r, 1100r, 806r, 514f, and 514r (71–75). The Staden (76) and the Geneious (77) software packages were used for trimming and aligning the forward and reverse sequences. The trimmed sequences were manually assessed and compared against the National Center for Biotechnology Information (NCBI) nonredundant nucleotide database (http://blast.ncbi.nlm.nih.gov) using the MegaBLAST algorithm (78) available on NCBI’s Basic Local Alignment Search Tool (BLAST) web interface (79) and the Ribosomal Database Project (RDP) (80). Identity was determined by database matches with E values of 0, identity of 99 to 100%, and 100% coverage. The global alignment of the nearly full-length 16S rRNA gene nucleotide sequences was performed using the ClustalW program (81), and phylogenetic analyses were performed using Molecular Evolutionary Genetics Analysis, version 6.0 (MEGA6), software (82). Phylogenetic trees were constructed using the neighbor-joining method (83), with distances being calculated using the Kimura 2-parameter method (84) and pairwise deletions of gaps. The phylogeny based on the 16S rRNA gene sequence was inferred using the maximum likelihood (ML) method (85). Bootstrap analysis with 10,000 replicates was used to assess the statistical strength of the branch positions (84). The 16S rRNA gene sequence from Methanobrevibacter ruminantium M1 (GenBank accession number CP001719) was used as an outgroup for the tree.

### Cell motility and flagellar biosynthesis operons.

To examine the motility of the *Butyrivibrio* strains, a motility agar stab test was carried out using 5 ml of 0.3% (wt/vol) agar in RM02 medium with cellobiose as the carbon source in a 10-ml Hungate tube (37). Freshly grown *Butyrivibrio* cultures were stab inoculated into the agar using a straightened inoculating loop, and the cultures were incubated at 37°C for 24 to 48 h. Nonmotile strains displayed visible growth confined to the area immediately surrounding the initial inoculation stab. Motile bacterial cells migrated through the agar to produce a diffuse or cloudy growth pattern, as evidenced by turbidity located a distance away from the inoculation stab (86). The strains used as negative controls included B. hungatei MB2003 (52), B. proteoclasticus B316^T^ (87), Prevotella ruminicola 23 (88), and Streptococcus bovis 2B (89), while P. xylanivorans MA3014 (37, 50) was used as a positive control. The variable regions of the flagellum biosynthesis operon were compared in *Butyrivibrio* strains and correlated with their motility in order to determine the relationship between the genotype and phenotype (90). Annotations from the Integrated Microbial Genomes with Metagenomes (IMG/M) system (91) were used to validate the functionality of the genes within the flagellum biosynthesis operons. The gene sequences were manually assessed using BLASTn from the BLAST+ package (92).

### Carbohydrate source utilization and fermentation end product analysis.

Bacterial carbon source utilization was tested on fresh *Butyrivibrio* cultures inoculated into Hungate tubes containing RM02 medium broth with the separate addition of each of the 32 carbon sources, including seven polysaccharides (glycogen, pectin, inulin, cellulose, dextrin, starch, and xylan) at a 0.5% (wt/vol) final concentration (37, 50). Cultures were inoculated in triplicate and grown under anaerobic conditions overnight at 39°C. The optical density at 600 nm (OD_600_) readings were measured initially after inoculation and after 24 h and 72 h of incubation. For the soluble substrates, changes in the OD_600_ (ΔOD_600_) readings of 0.5 to 1.0 were scored as ++, changes of 0.2 to 0.5 were scored as +, and changes of 0 to 0.2 were scored as −. Positive controls were individual strains grown in cultures containing d-glucose, d-cellobiose, d-xylose, and l-arabinose. Two types of negative controls were included: inoculation controls without a carbon source added and uninoculated medium with the substrate. Uninoculated medium without the substrate was used as a blank. Growth on polysaccharide substrates was assessed by measurement of volatile fatty acid (VFA) production. VFA production was determined from triplicate broth cultures grown overnight with cellobiose as the substrate and analyzed for formate, acetate, propionate, *n*-butyrate, isovalerate, and lactate on an HP 6890 series gas chromatograph (Hewlett-Packard) with 2-ethylbutyric acid (Sigma-Aldrich) as the internal standard. To derivatize formic, lactic, and succinic acids, samples were mixed with the HCl ACS reagent (Sigma-Aldrich) and diethyl ether, with the addition of *N*-methyl-*N-t*-butyldimethylsilyltri-fluoroacetamide (MTBSTFA) (Sigma-Aldrich) (93). A d-lactic acid assay kit and l-lactic acid assay kit (Megazyme Inc., Bray, Ireland) were used for measurements of d- and l-lactate concentrations, respectively. All samples were diluted to yield a lactic acid concentration of 0.03 to 0.30 g/liter, the linear range of the assay. The microplate assay procedure was performed according to the manufacturer**’**s instructions with a 224-μl reaction volume.

### Screening for enolase genes.

For comparisons of enolase gene (*eno*)-positive versus enolase gene-negative *Butyrivibrio* strains, the *eno* genes were identified and annotated based on the Integrated Microbial Genomes (IMG) system of identification of enolase Pfam (C-terminal Pfam00113 and N-terminal Pfam03952), COG (COG0148), KOG (KOG2670), and KO (KO1689) domains. In addition, the Metastats program (94) was employed in conjunction with contrasting upper and lower quartile or percentile gene counts, in order to identify additional functions with a pattern of preservation/loss similar to that of the glycolytic enolase gene (7). The *eno* gene Pfam domains were compared for enolase-positive *Butyrivibrio* strains, and the respective amino acid sequences of the enolase proteins were compared using a maximum likelihood (ML) alignment analysis. Genomic DNAs from a selection of *Butyrivibrio* strains were extracted as described above and screened for the presence of enolase genes by PCR amplification using the forward primer 5′-AATGGACCTAYGCAGATGC-3′ and reverse primer 5′-ATCTGGTTRAGCTTWATAAG-3′ (49). The PCR cycling conditions used were 94°C for 2 min, followed by 35 cycles of 94°C for 30 s, 50°C for 30 s, and 72°C for 2 min and a final extension time of 10 min at 72°C. The PCR products were analyzed by agarose gel electrophoresis, and the concentrations were determined with a NanoDrop spectrophotometer and a Qubit double-stranded DNA BR assay kit (Thermo Fisher Scientific Inc.). To investigate the possibility that the enolase primers did not detect all enolase genes, the enolase primer sequences were aligned against the draft genomes of the *Butyrivibrio* strains. A strong alignment of both the forward and the reverse degenerative primers was achieved for all enolase-positive *Butyrivibrio* strains that screened positive for presence of *eno*.

### Sequence assembly and annotation.

All Hungate genomes were sequenced at the DOE Joint Genome Institute (JGI) using the Illumina technology (95) or Pacific Biosciences (PacBio) RS technology (96). For all genomes, we either constructed or sequenced an Illumina short-insert paired-end library with an average insert size of 270 bp or a PacBio SMRTbell library. Genomes were assembled using the Velvet (97), ALLPATHS (98) or Hierarchical Genome Assembly Process (HGAP) (99) assembly methods. Genomes were annotated by the DOE JGI genome annotation pipeline (100, 101). Briefly, protein-coding genes (coding sequence [CDSs]) were identified using the Prodigal program (102), followed by a round of automated and manual curation using the JGI GenePrimp pipeline (103). Functional annotation and additional analyses were performed within the Integrated Microbial Genomes Expert Review (IMG-ER) platform (91).

### Comparative analysis of the genome data sets.

**(i) CAZyme annotation.** The putative proteomes of the 40 *Butyrivibrio* and 6 *Pseudobutyrivibrio* data sets were subjected to automated annotation and assignment to CAZymes using the dbCAN resource CAZy family-specific hidden Markov models (HMMs) (104). An E value of <1e^−3^ for CAZymes based on family-specific HMMs was used as the cutoff for alignments shorter than 80 amino acids, while an E value of <1e^−5^ was used for alignments longer than 80 amino acids. These cutoff settings enabled short but significant CBM matches to be maintained. All dbCAN hits were clustered at a 100% sequence identity threshold using the CD-HIT Illumina algorithm to remove duplicates (105). All descriptions and classifications were compiled from CAZy (106), and the modular architectures of CAZymes and predicted proteins with multimodular CAZyme organizations in the genome data sets were determined by searching each query protein against the Pfam and Protein Data Bank (PDB) databases (107, 108).

**(ii) ANI and AF computation.** ANI and the fraction of orthologous genes (AF) were used as complementary measures of genetic relatedness based on the gene content between the 40 *Butyrivibrio* and 6 *Pseudobutyrivibrio* genomes. ANI is a measure of nucleotide-level genomic similarity between the coding regions of two genomes, determined using a custom Perl script and the high-performance similarity search tools NSimScan and PSimScan (109). Each genome sequence served as a reference genome, and the resulting ANI values were averaged. The code to perform genomic ANI and AF computation is available at https://ani.jgi.doe.gov/html/download.php. The AF and ANI were calculated for the 40 *Butyrivibrio* and 6 *Pseudobutyrivibrio* genomes to determine species cutoffs. In order to identify species ANI and AF that determine whether the genomes in a pair belong to the same species, only the subset of high-quality genome pairs was utilized. An ANI cutoff of ≥96.5 and an AF cutoff of ≥0.6 were used to define species.

**(iii) FGD.** The functional genome distribution (FGD) is a tool for comparative microbial genomics analysis and interpretation of the genetic diversity of bacteria (110). FGD investigates the overall similarity levels between microbial genomes, based on the amino acid sequences of their predicted ORFeomes, which correspond to the coding sequences (CDSs) of the genes (open reading frames [ORFs]) in a genome, and ultimately defines the degree of similarity of the genomes. All Hungate *Butyrivibrio* and *Pseudobutyrivibrio* genomes were downloaded in FASTA format from the IMG genome database (111), concatenated using a universal spacer-stop-spacer sequence, and automatically annotated using the GAMOLA2 software package (112). The in-house closed genomes of B. proteoclasticus B316^T^ (49) and B. hungatei MB2003 (52) and the draft genome of P. xylanivorans MA3014 were manually annotated using GAMOLA2 (112). The predicted ORFeomes of all genomes were subjected to an FGD analysis, and the resulting distance matrix was imported into MEGA6 (82). The functional genome distribution was visualized using the unweighted pair group method with arithmetic mean (UPGMA) method (113, 114).

**(iv) Genome alignment.** A MUMmer (version 3) system was used to compare the alignment of contigs across a reference genome in the form of a dot plot diagram (115). Synteny plots were generated using the mummerplot utility. The plots reveal regions of exact matches between the pair of genomes compared and thus are an indicator of the conservation between the two genomes. The Gsview program (116) was used to visualize the generated MUMmer plot.

**(v) Amino acid and codon usage analyses.** A comparison of the amino acid and codon usage between the 40 *Butyrivibrio* and 6 *Pseudobutyrivibrio* genomes was performed using the CMG-biotools package (117) under the default parameters. Amino acid and codon usages were calculated using BioPerl modules, which calculate each amino acid or codon count as a fraction of the total count of amino acids or codons. The percent codon and amino acid usage was plotted in two-dimensional heat maps using gplots in R (118), reordering the organisms and the amino acids/codon to show the shortest distance between them. Dendrograms were used to visualize the difference in usage between different strains.

**(vi) Determination of the core and pan-genomes.** The genes representative of the *Butyrivibrio* and *Pseudobutyrivibrio* core and pan-genomes were determined by performing a BLAST-based analysis using the CMG-biotools package (117) with default parameters. If two proteins within a genome met the designated cutoff, they were clustered into one protein family. Protein families were extended via single-linkage clustering. If a protein family included proteins from all genomes in the comparison, the family was designated a core protein family. Subset genes, such as species group shared and unique subsets of genes within individual genomes, were identified by clustering the results from the core and pan-genome calculations.

### Data availability.

The data sets supporting the conclusions of this article are available through the IMG portal (https://img.jgi.doe.gov/). Additionally, a dedicated portal to download all genomes sequenced as part of the Hungate1000 project (7) is provided at https://genome.jgi.doe.gov/portal/pages/dynamicOrganismDownload.jsf?organism=HungateCollection.

## Supplementary Material

Supplemental file 1

Supplemental file 2

Supplemental file 3
