# Supplementary material for: Comparative Genomics of Rumen Butyrivibrio spp. Uncovers a Continuum of Polysaccharide-Degrading Capabilities
Source: Appl Environ Microbiol. 2019 Dec 13;86(1):e01993-19. doi: 10.1128/AEM.01993-19 (PMC6912079; doi:10.1128/AEM.01993-19)
Supplement: Supplemental file 1 [file AEM.01993-19-s0001.pdf]

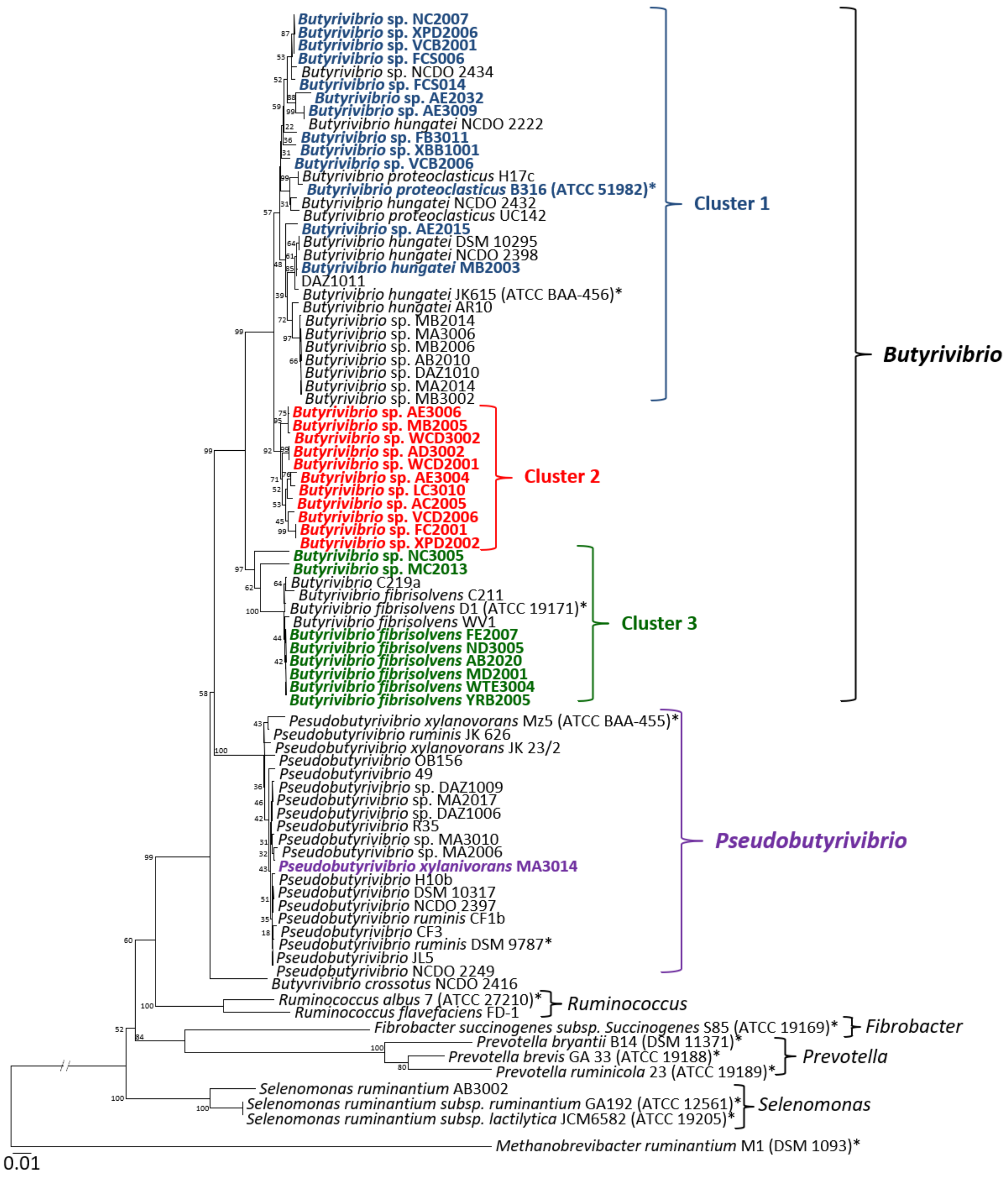

Figure S1. Phylogenetic tree of *Butyrivibrio* strains based on 16*S* rRNA full-length gene sequence data. Bar, indicates 0.01 nucleotide substitutions per site or 1% difference in nucleotide sequence. * indicates type strains. Strains shown in bold and coloured were selected for further characterisation.

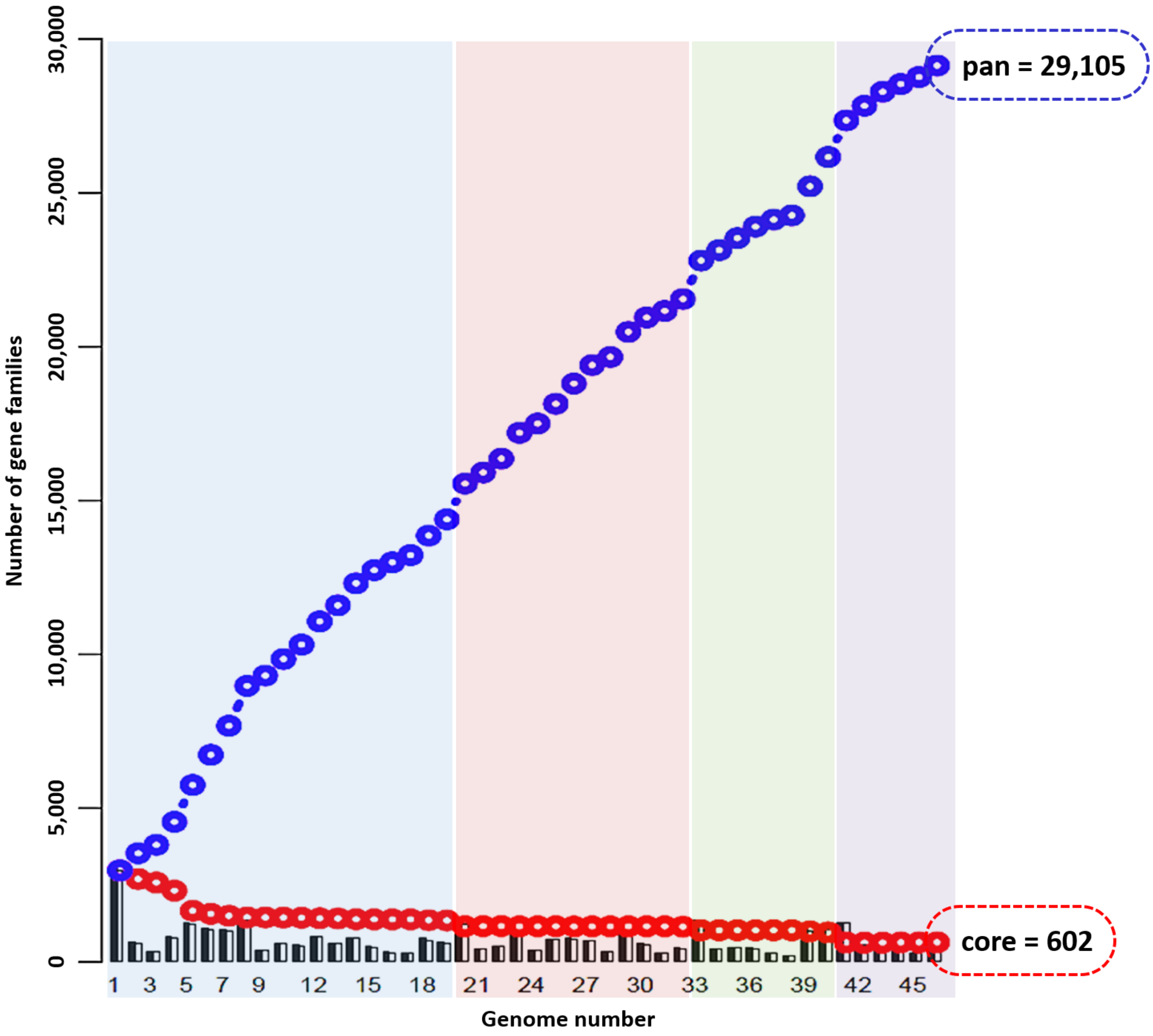

Figure S2. Core- and pan-genomes of *Butyrivibrio* and *Pseudobutyrivibrio* defined using BLAST analysis. The number of gene families belonging to the pan-genome are shown as blue circles and the core-genome shown in red circles. Black bars, number of new genes in genome; white bars, number of new gene families in genome. Genome numbers correspond to the order of genomes in Data Set S1.

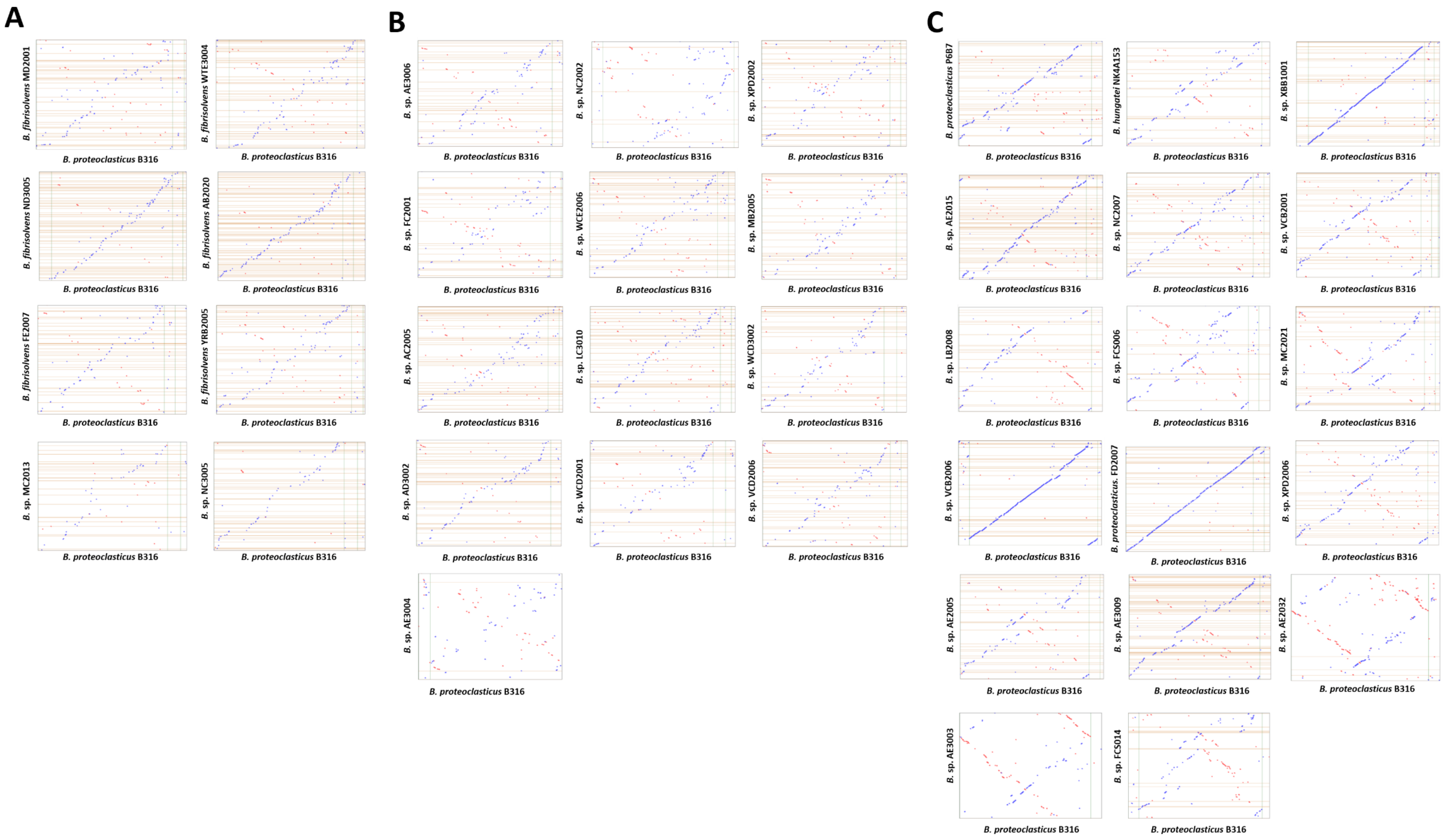

Figure S3. MUMmer plots of *Butyrivibrio* draft genomes versus *B. proteoclasticus* B316^{T} as the reference genome. A, *Butyrivibrio* Cluster 3 strains. B, *Butyrivibrio* Cluster 2 strains. C, *Butyrivibrio* Cluster 1 strains. Colour codes: blue, forward sequence, red, reverse sequence.

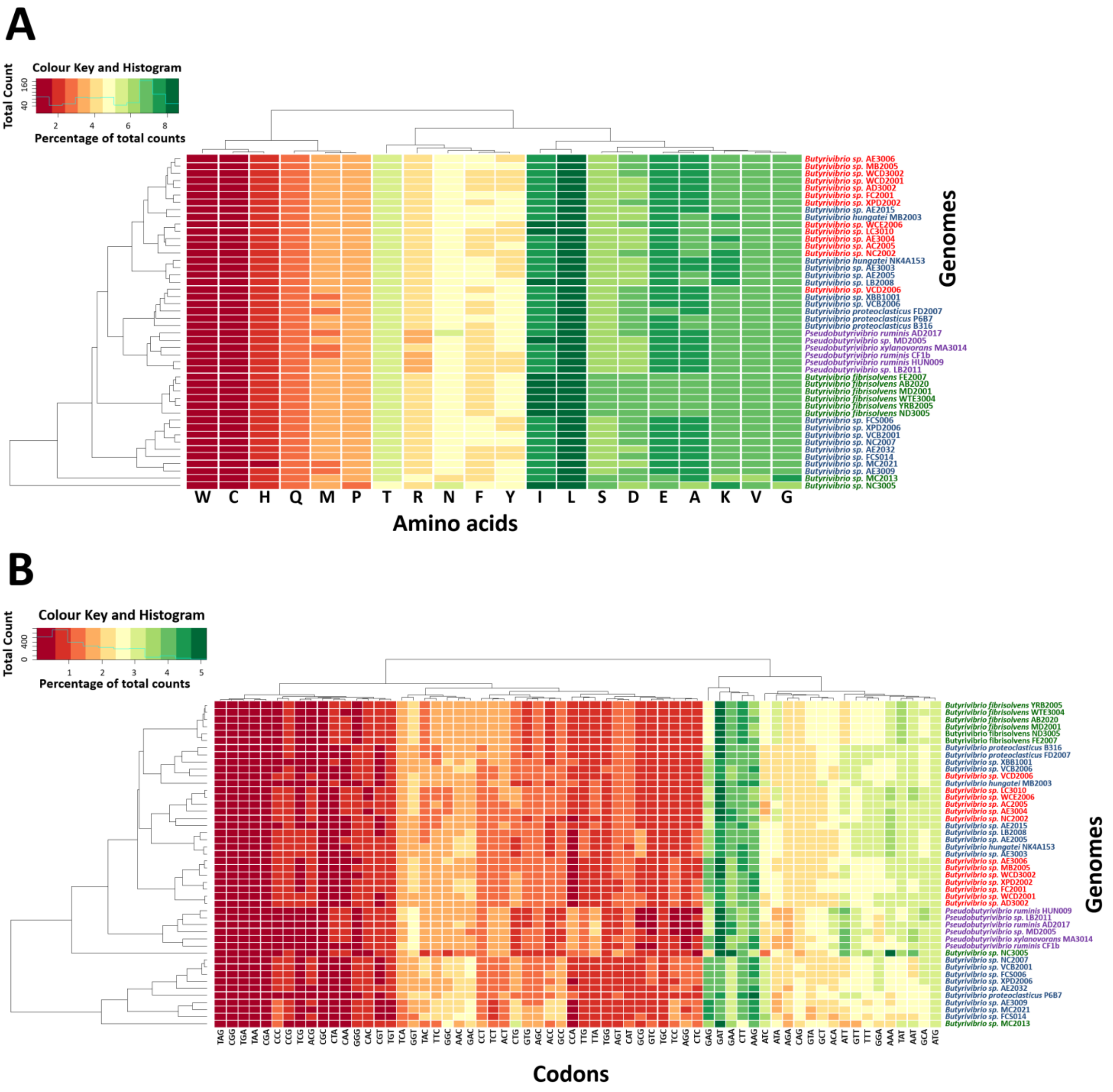

Figure S4. Amino acid and codon usage of *Butyrivibrio* and *Pseudobutyrivibrio* draft genomes based on their protein content. A, Amino acid usage heatmap. B, Codon usage heatmap. Cluster grouping was based on comparison of the ORFeomes of 46 genomes by FGD analysis. Genomes are coloured to represent *Butyrivibrio* Cluster 3 in green, *Butyrivibrio* Cluster 2 in red, *Butyrivibrio* Cluster 1 in blue and *Pseudobutyrivibrio* in purple.

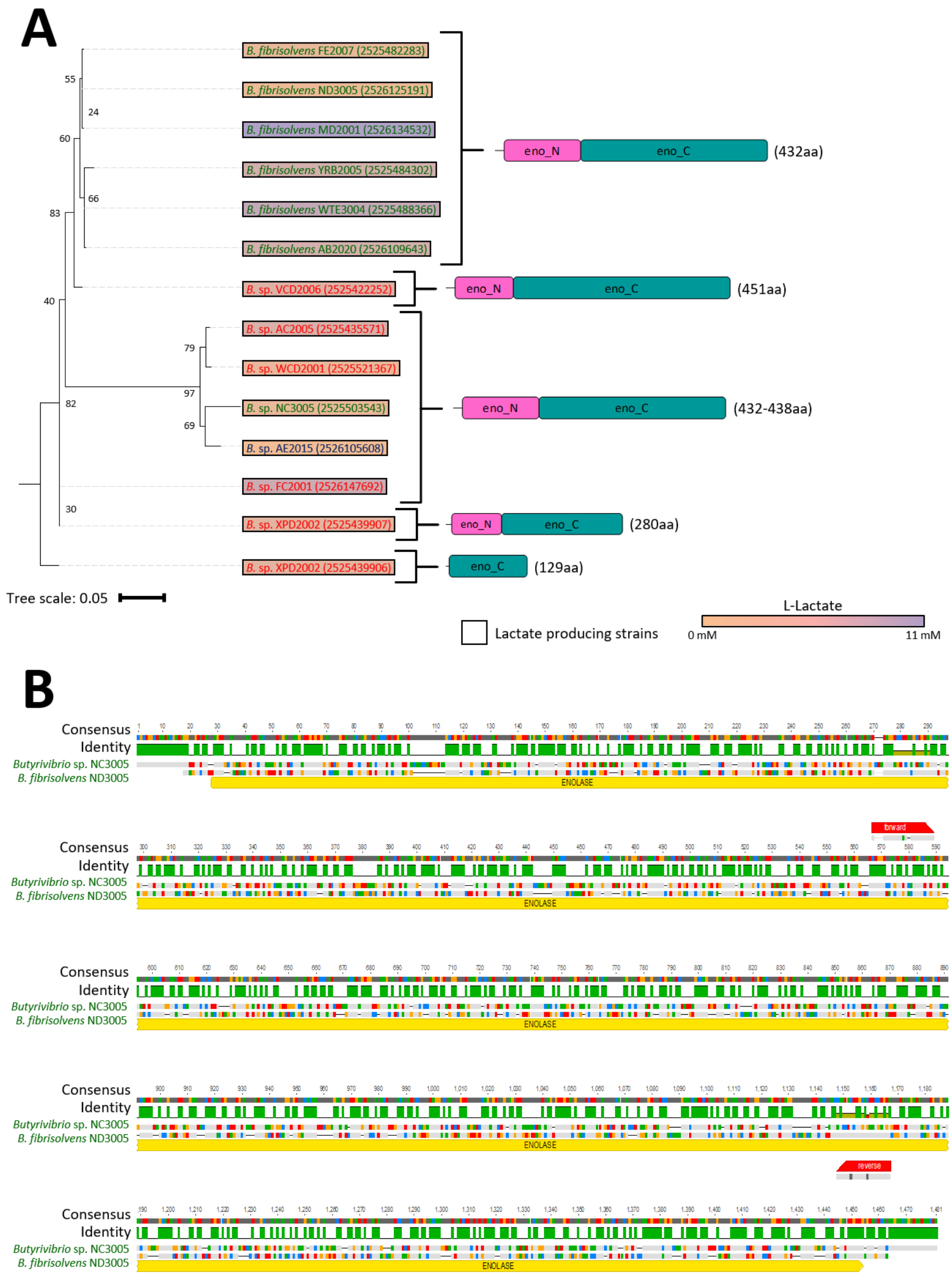

Figure S5. Enolases from *Butyrivibrio* genome sequences. A, Maximum Likelihood analysis and comparison of Pfam domains for enolase proteins and genes, respectively. The scale indicates the number of aa substitutions per site. The analysis involved 14 amino acid sequences and there were a total of 454 positions in the final dataset. Strains are coloured to represent *Butyrivibrio* Cluster 3 in green, *Butyrivibrio* Cluster 2 in red, *Butyrivibrio* Cluster 1 in blue. All *Butyrivibrio* strains produced exclusively L-lactate. Abbreviations: eno_N, enolase N-terminal domain (Pfam03952); eno_C, enolase, C-terminal TIM barrel domain (Pfam00113). B, genomic nucleotide alignment of the enolase gene containing the primer binding sites. Enolase gene shown in Cluster 3 *eno*$^{+}$ *B. fibrisolvens* ND3005 (positive *eno* PCR product) and predicted *eno*$^{+}$ *Butyrivibrio* sp. NC3005 (absence of an *eno* PCR product) strains.
